# First sex modification case in equine cloning

**DOI:** 10.1371/journal.pone.0279869

**Published:** 2023-01-04

**Authors:** Mariana Suvá, Victoria Helga Arnold, Elisabet Astrid Wiedenmann, Roberto Jordan, Emanuel Galvagno, Marcela Martínez, Gabriel Damián Vichera

**Affiliations:** 1 Kheiron Biotech S.A., Pilar, Buenos Aires, Argentina; 2 Laboratorio de Genética Aplicada, Sociedad Rural Argentina, Buenos Aires, Argentina; University of Massachusetts Amherst, UNITED STATES

## Abstract

Somatic cell nuclear transfer (SCNT) is an asexual reproductive technique where cloned offspring contain the same genetic material as the original donor. Although this technique preserves the sex of the original animal, the birth of sex-reversed offspring has been reported in some species. Here, we report for the first time the birth of a female foal generated by SCNT of a male nuclear donor. After a single SCNT procedure, 16 blastocysts were obtained and transferred to eight recipient mares, resulting in the birth of two clones: one male and one female. Both animals had identical genetic profiles, as observed in the analysis of 15-horse microsatellite marker panel, which confirmed they are indeed clones of the same animal. Cytogenetic analysis and fluorescent *in situ* hybridization using X and Y specific probes revealed a 63,X chromosome set in the female offspring, suggesting a spontaneous Y chromosome loss. The identity of the lost chromosome in the female was further confirmed through PCR by observing the presence of X-linked markers and absence of Y-linked markers. Moreover, cytogenetic and molecular profiles were analyzed in blood and skin samples to detect a possible mosaicism in the female, but results showed identical chromosomal constitutions. Although the cause of the spontaneous chromosome loss remains unknown, the possibility of equine sex reversal by SCNT holds great potential for the preservation of endangered species, development of novel breeding techniques, and sportive purposes.

## Introduction

Somatic cell nuclear transfer (SCNT) is an asexual reproductive technique commercially available in horses. It is mainly applied to multiply elite individuals [1, 2], but also to reintroduce valuable genomic backgrounds to breeding programs in cases where the original horse has died or is no longer fertile [3]. General interest in SCNT technology is based on the fact that cloned offspring contain the same genomic information as the original donor. However, genomic alterations have been sporadically found in clones of some species. Some of the most striking examples are related to sex modification in the cloned offspring. In the mouse, a female offspring was born from a male nuclear donor as a consequence of the spontaneous loss of the Y chromosome [4]. In dogs, incomplete epigenetic reprogramming after SCNT led to a hypermethylated Y chromosome, resulting in genetically male (XY) animals with a female phenotype [5, 6]. A similar result was reported in wolves, although the cause of sex reversal was not defined [7]. In horses, and despite the numerous clones born to date, no sex-associated genetic alterations have been described to the best of our knowledge. Here, we report a viable female horse born by SCNT of a male nuclear donor. This is the first report of sex modification in equine cloning, which opens the door to sex manipulation by SCNT in this species.

## Results

**Fig 1:**
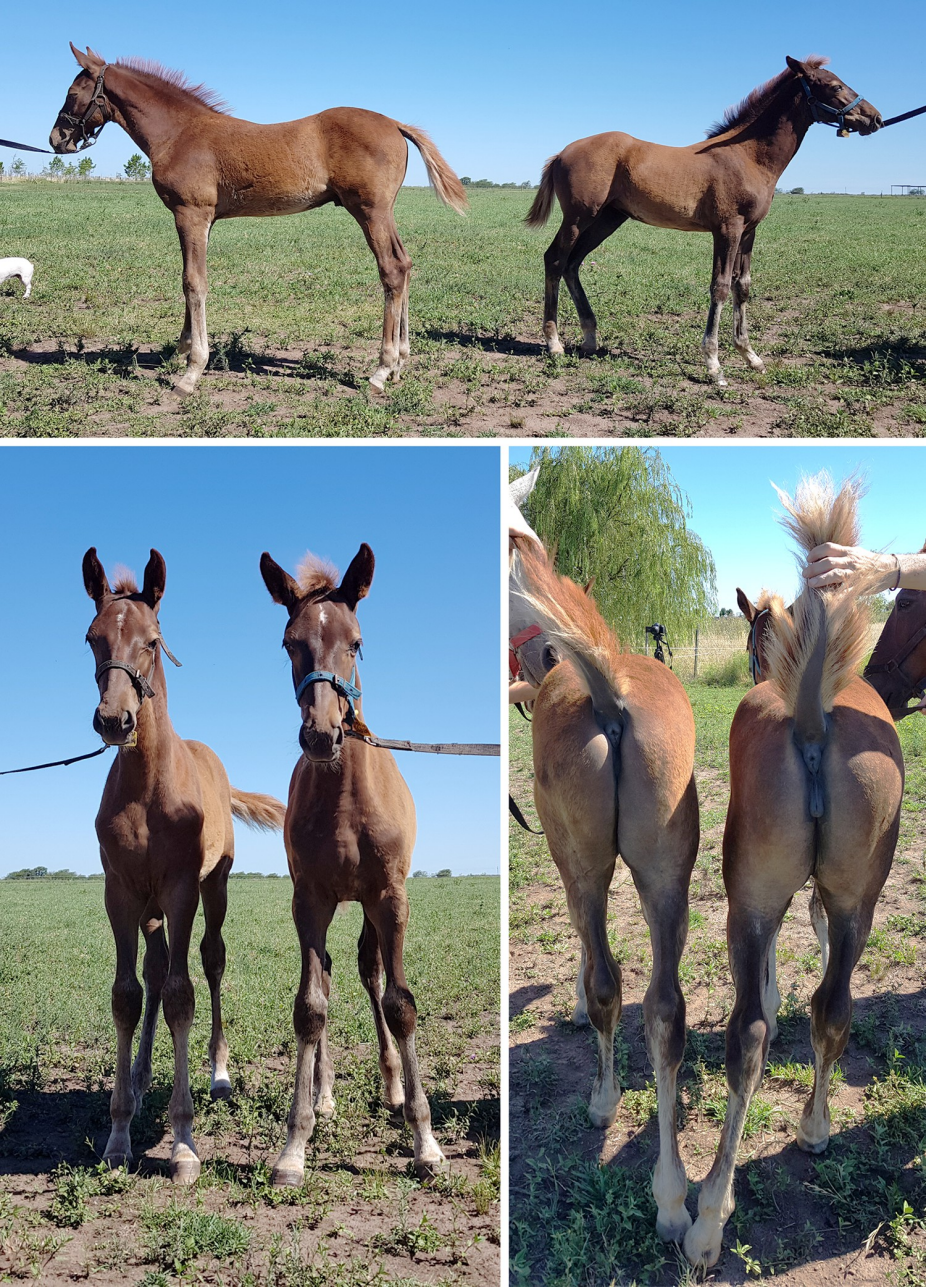
Foals born after a SCNT procedure using male-horse-derived MSCs as nuclear donors. Male: left, female: right.

Passage-2 mesenchymal stem cells (MSCs) derived from a male horse were used as nuclear donors for a standard SCNT procedure performed in November 2019 (Table 1). Two live foals were born in November 2020, one male–as expected, considering the sex of the nuclear donor–and one female (Fig 1). At the time of birth, the female foal showed no anatomic abnormalities, although internal reproductive structures could not be observed yet given the animal’s young age. At the age of 2 years, it still shows normal external genitalia, with a normally sized and shaped vulva and clitoris. Ultrasonographic evaluation was performed, and showed a normal uterus but small underdeveloped ovaries (right: 10 x 6 mm; left: 7 x 5 mm), suggesting gonadal hypoplasia. In addition, there was no sign of corpora lutea.

**Table 1:** *In vitro* and *in vivo* development of embryos obtained after a SCNT procedure using MSCs of a male donor.

Oocytes	Mature oocytes (%)	Embryos	Cleavage (%)	Blastocysts (%)	No. of recipients	No. of transferred embryos	Pregnancies (%)	Born (%)	Days of gestation	Sex
420	225 (53.57)	170	125 (73.53)	17 (10)	8	16	3 (37.5)	2 (25)	366/351	male/female

### Genotyping of the born foals

**Fig 2:**
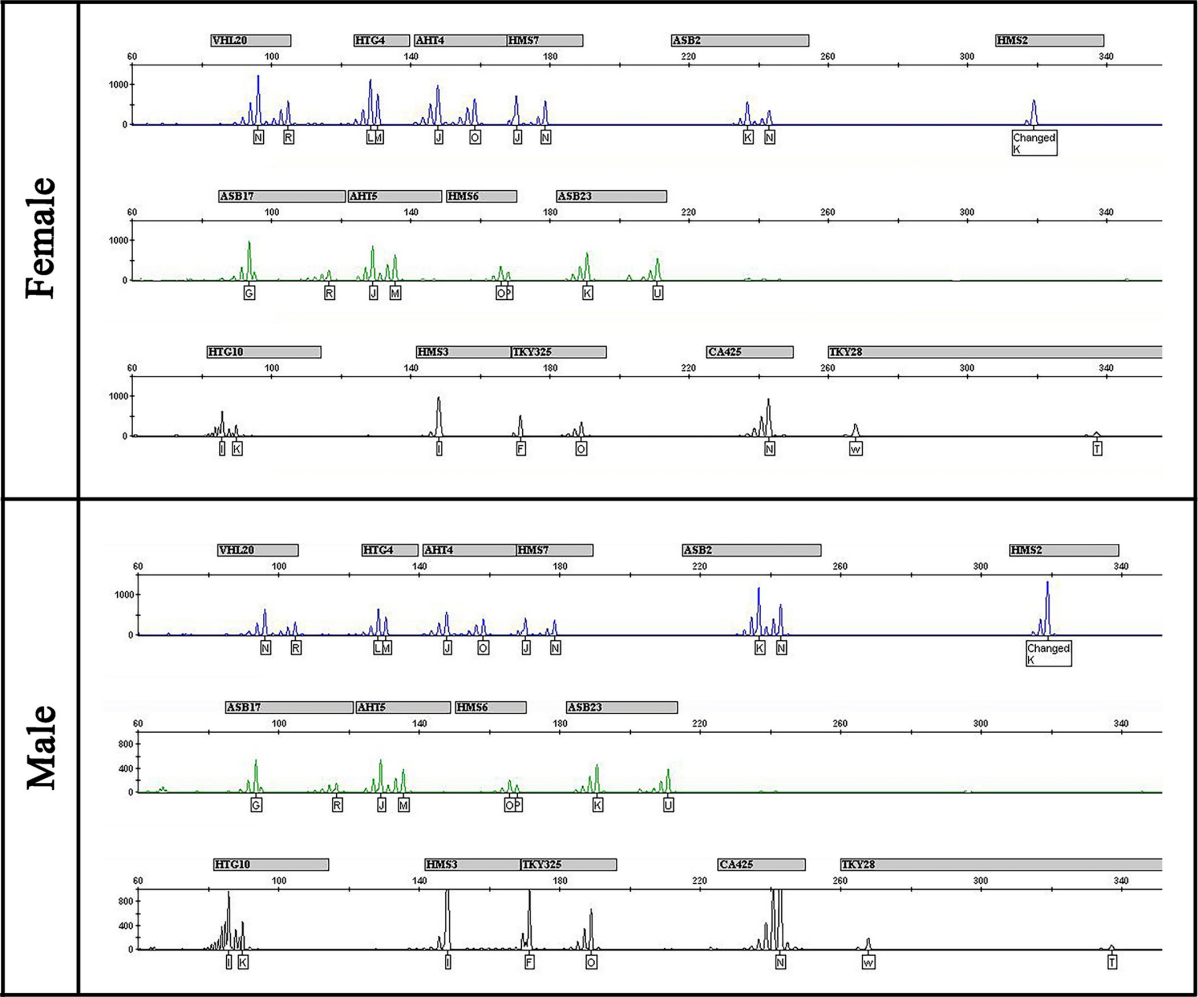
Microsatellite profiles from the foals. Results from the analysis of a 15-panel of microsatellites, used for routine parentage and identity purposes.

In order to determine whether the two born clones are indeed genetically identical, hair samples from each foal were genotyped with a 15-horse microsatellite marker panel (Fig 2). Analyses rendered identical genotypes, which showed that both foals are indeed clones derived from the same donor. To evaluate possible mosaicism in the female clone, a blood sample was also analyzed, with results showing the same profile in both hair and blood samples.

### Cytogenetic and molecular analysis of the sex chromosomes

**Fig 3:**
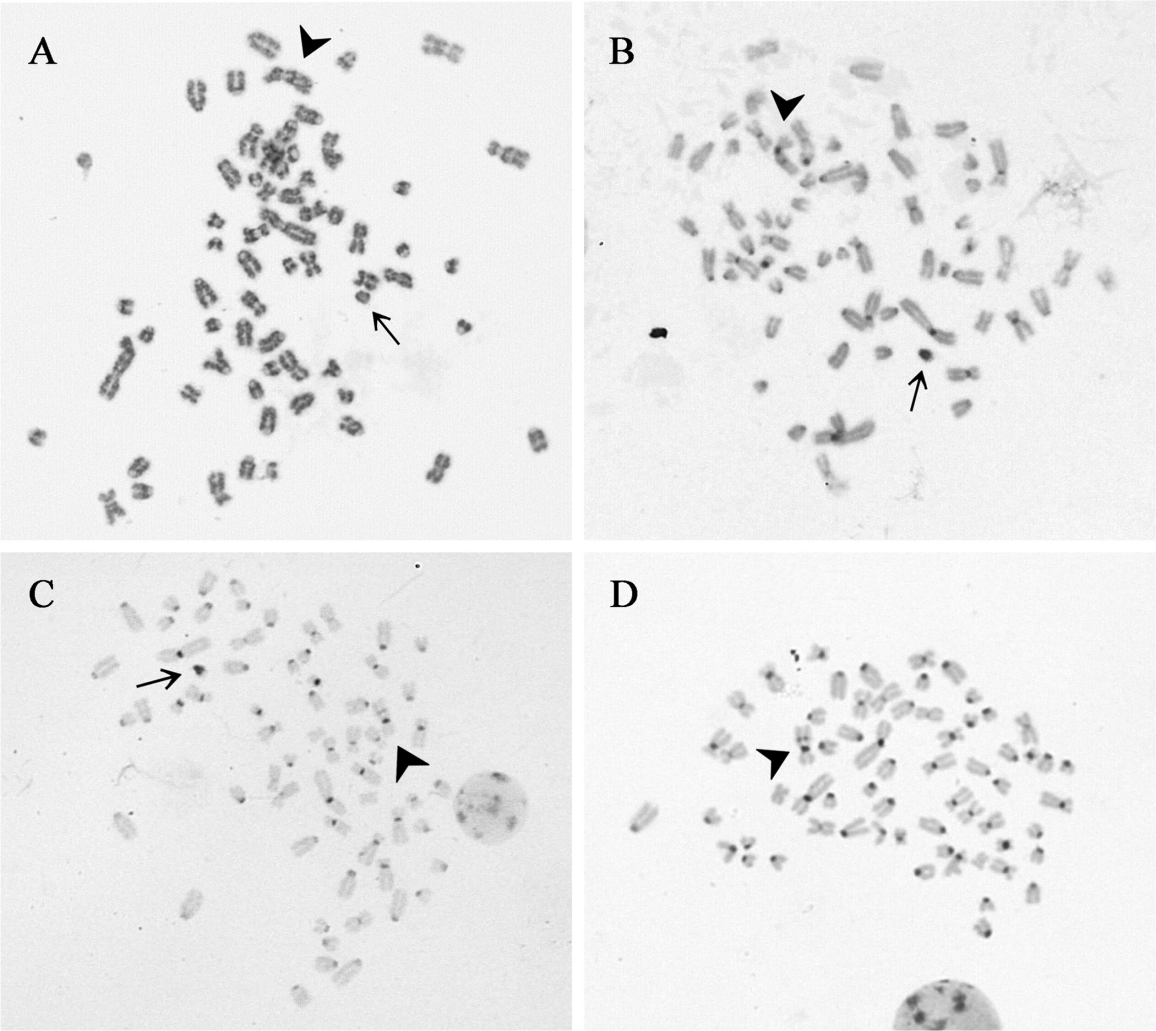
Representative metaphase spreads from the foals and the donor MSCs. (A) G banding and (B) C banding from the MSCs used as nuclear donors (64,XY). (C) C banding from the male clone (64,XY). (D) C banding from the female clone (63,X). Arrows indicate the Y chromosome and arrowheads indicate the X chromosome. Magnification 1000X.

**Fig 4:**
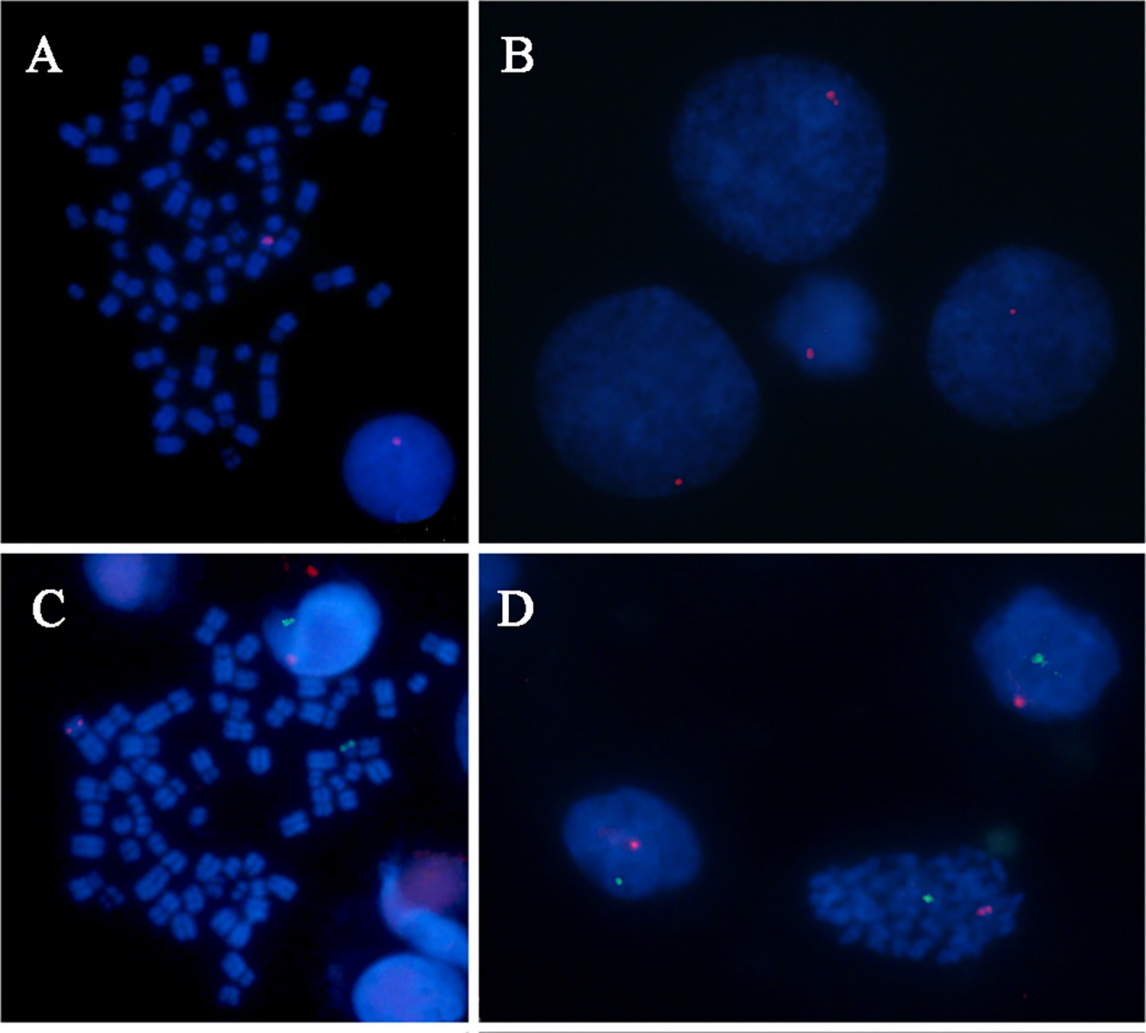
Specific identification of the X and Y chromosomes by FISH. (A) and (B) Blood cells from the female foal. (C) Metaphase spread derived from the blood sample of the male foal. (D) Interphase cells from the MSC culture used as nuclear donor. Red color indicates the X chromosome probe and green color indicates the Y chromosome probe. Magnification 1000X.

After confirming that the female and male foals are clones, cytogenetic analyses were carried out to elucidate the cause of this sex modification. Fibroblast (n = 30) and blood samples (n = 100) obtained from the female and male clones were evaluated by conventional Giemsa staining, C banding and G banding to determine the chromosome number and sex chromosome constitution. In addition, the MSCs used as nuclear donors (n = 80) were also analyzed. The MSCs (Fig 3A and 3B) and the samples from the male foal (Fig 3C) showed the expected 64,XY chromosome set, whereas the samples from the female foal presented a 63,X chromosome set (Fig 3D). In addition, fluorescence *in situ* hybridization (FISH) using X and Y specific probes on the MSCs (n = 268) and blood samples from the male (n = 278) and female (n = 309) clones confirmed the X monosomy in the female foal (Fig 4A and 4B), and the normal male sex chromosome configuration in the male foal and donor MSCs (Fig 4C and 4D, respectively).

**Fig 5:**
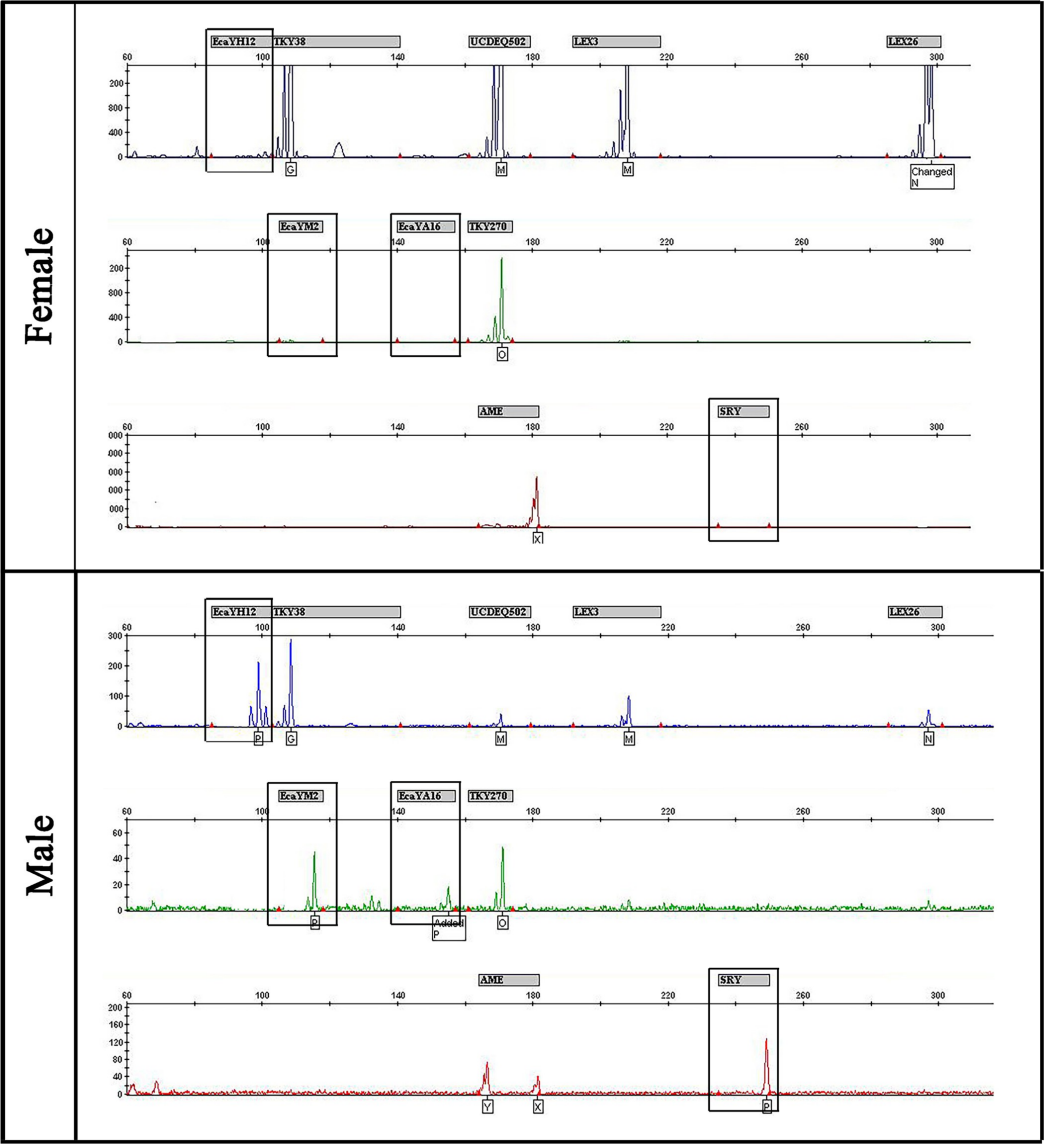
Characterization of X- and Y-linked markers. Results of microsatellite analysis in the female and male horses. Boxes indicate the Y-linked markers.

Moreover, PCR analysis of X- and Y-linked markers was performed using genomic DNA derived from hair samples of the male foal, and hair and blood samples of the female foal (Fig 5). The sample from the male foal amplified the *AMEL-X* and *AMEL-Y* chromosome markers, whereas the samples from the female foal amplified only the *AMEL-X* chromosome marker. In addition, the female clone showed the profile expected for a female genotype, without amplification of the *SRY* gene or any of the Y-linked short tandem repeats (STR) markers. X-linked STRs in the female and male clones showed homozygous genotypes, as expected for genotypes carrying only one X chromosome.

## Discussion

In SCNT, sex is determined by the genotype of the nuclear donor. However, alterations involving the sex chromosome pair have been found in clones of some species. Here, we report the first sex modification in cloned horses, opening the door to sex manipulation through SCNT in this species.

Cytogenetic and molecular analysis of the female foal showed that the sex modification was the consequence of a Y chromosome loss that led to an X monosomy. This alteration might have occurred in the cell culture, or during early embryo development. Previous studies report a 10.2% incidence of X monosomy on mouse established XX and XY embryonic stem cell cultures [8], and around 15–18% of aneuployd cells in passage 2 human MSC cultures [9]. Different factors are thought to induce alterations in the chromosome number of cells in culture, including passage number [9, 10], *Mycoplasma* infection [11] and stress [12]. The MSCs used as nuclear donors in this work were at passage 2, and *Mycoplasma* contamination is periodically evaluated in our lab. Therefore, these factors were excluded as possible causes of chromosomal alterations. Nevertheless, cell samples were maintained at ambient condition for several hours before SCNT. According to the literature, the room temperature could have induced a stress response [13], which led to genome instability. In addition, the high oxygen tension triggers an oxidative stress response in MSC cultures as a consequence of a high oxygen consumption rate, which is strongly related to DNA damage and unstable chromosomal aberrations [9]. Nevertheless, the MSCs used as nuclear donors contained an euploid chromosome count according to the cytogenetic analysis. Alternatively, a spontaneous chromosome loss may have occurred during the early embryonic stages. In this case the result would be a mosaic embryo and, consequently, a mosaic female foal with 63,X and 63,XY cells. However, the two tissues evaluated in this study–blood and skin, which represent two different embryonic layers–showed the same genetic constitution according to karyotyping and PCR genotyping. Therefore, this alteration occurred in the cell culture at a rate below 1/348 and could not be detected, the female is indeed mosaic and presents 63,XX cells in tissues different from blood and skin, or the Y chromosome was lost during the earliest embryonic stage.

The horse is an attractive model for sex manipulation, as the X0 sex chromosome condition is well tolerated in this species. In fact, horses present the second-highest incidence, only after humans, of viable X chromosome monosomy. The negative correlation between the incidence of viable X0 individuals and the size and gene content of the pseudoautosomal region (PAR) of the sex chromosomes suggests that horses present a permissive genetic background towards this chromosomal alteration compared to other species [14].

Different strategies can be evaluated in horses to specifically eliminate a sex chromosome, according to previous reports in other species. For example, human chromosome 21 was functionally eliminated by insertion of a XIST transgene, which led to a stable chromatin modification and the silencing of the extra chromosome in human Down syndrome pluripotent stem cells [15]. In addition, targeted chromosome elimination was achieved through the insertion of oppositely oriented *loxP* sites and Cre-mediated sister chromatid recombination [16], or through the insertion of a TKNEO transgene into a targeted chromosome followed by selection of cells that spontaneously lost the edited chromosome [17]. More recently, the complete Y chromosome has been deleted using CRISPR/Cas9 technology in the mouse [18]. Furthermore, sex-reversal has been achieved in mice [19], rabbits [20], and pigs [21] through SRY gene knock-out, which yielded phenotypic females with an XY genotype. However, the use of these strategies involves gene editing techniques that would require approval from regulatory entities.

Alternatively, chromosome instability can be induced in cell cultures to promote spontaneous chromosome losses as described above while avoiding the use of molecular tools. Derivation of clonal cell lines, as achieved previously by our group in horses [22], may enable the isolation, identification, and replication of the X0 cells. Therefore, establishing X0 cell cultures, which might additionally be combined with specific X or Y chromosome transfer to zygotes [23, 24], could result in sex modification in horses by SCNT.

Equine sex manipulation can be applied to the preservation of endangered species, Turner syndrome research, and reproductive and sportive purposes. To date, conservation breeding programs are the main strategy applied for the preservation of wild horses. However, advanced assisted reproductive technologies (ARTs) are a powerful tool to be included in conservation programs for endangered species [25–27]. Derivation of X0 cell lines combined with specific sex chromosome transfer and SCNT might enable the production of individuals of both sexes from cryopreserved samples of endangered and even extinct species. The horse stands as a particularly attractive model in this case, as the genetic plasticity shared among *Equus* [27] could enable the sex modification of individuals using X or Y chromosomes from a different equid species. Regarding medical research, 63,X mares show phenotypic features similar to those of Turner syndrome in humans, unlike those found in X0 mice [14], which makes the horse an attractive model for the study of this syndrome. Moreover, sex manipulation could be used as a breeding tool. If a female clone can be derived from a male horse and then bred, the genetic material from two original males could be combined. Finally, regarding commercial application, sex modification could be applied to sporting individuals. Polo, for example, is a sport practiced worldwide for which female horses are preferred over males. Considering that advanced biotechnologies are popular within this equine discipline (see the Argentine Polo Pony Breeder’s Association Statute at www.criapoloargentino.com.ar), obtaining a female clone from an exceptional male player may be of interest.

In conclusion, although the cause of the spontaneous chromosome loss reported in this work and how to intentionally reproduce it in the laboratory remain to be elucidated, the present report shows that sex manipulation by SCNT is feasible in horses, a finding of great potential for the preservation of endangered species, development of novel breeding techniques, and sportive purposes.

## Materials and methods

### Animal care and use of research animals

This study was carried out following the Guide for the Care and Use of Agricultural Animals in Agricultural Research and Teaching. The protocols involving animal manipulations were approved by the Institutional Committee for the Care and Use of Experimental Animals of the San Martin National University, Buenos Aires, Argentina (CICUAE-UNSAM, Permit Number: 001/16).

### Isolation and culture of MSCs

MSCs were obtained from bone marrow aspirates of an adult male as described previously [2] with slight modifications. Briefly, aspiration was performed under sedation using 1 mL acepromazine maleate (Holliday-Scott SA, Buenos Aires, Argentina) and local anesthesia with 2% lidocaine. By pressing on the sternum, 200–300 ml of bone marrow aspirate was collected into a blood bag treated with sodium citrate. After discarding the blood through an infusion guide and washing with PBS, MSCs were collected through enzymatic treatment of the filter and blood bag with 0.25% Trypsin-EDTA (25200–56; Gibco, Waltham, MA, US) at 37°C for 10–15 min and vigorously shaking the bag and filter of the infusion guide. Finally, cells were collected by washing twice with 25 ml of DMEM-high glucose (10569–010; Gibco, Waltham, MA, US) supplemented with 20% fetal bovine serum (FBS, 10499–044; Gibco, Waltham, MA, US), 2% antibiotic–antimycotic (ATB; penicillin, streptomycin, and amphotericin B; 15240–096; Gibco), 1 μL/ml insulin-transferrin-selenium (ITS; 51300–044, Gibco) and 10 μl/ml MEM non-essential amino acids (11140–050; Gibco, Waltham, MA, US) and plated into a 150 mm cell culture dish (430599, Corning). Culture medium was changed twice a week until culture reached 70–80% confluence, when cells were cryopreserved in DMEM-high glucose supplemented with 20% FBS and 10% DMSO. At the moment of shipping this samples in particular, cells were thawed and plated on T25 flasks at 30% confluence, where they were kept for around 55 h at room temperature during transportation. When cells were received at the laboratory, flasks were immediately put at 38°C and 5% CO2 until 80% confluence, when cells were cryopreserved as described above.

### SCNT and embryo transfer

The cloning procedure was performed as described previously [1] with some modifications. Briefly, equine oocytes were *in vitro* matured in HEPES-buffered TCM-199 (12340–030; Gibco) supplemented with 10% FBS, 1 μl/ml ITS, 1 mM sodium pyruvate (P2256), 100 mM cysteamine (M-9768), 0.1 mg/mL of follicle-stimulating hormone (NIH-FSH-P1, Folltropin1; Bioniche, Belleville, ON, Canada), and 2% ATB at 38°C. Cumulus cells were removed by vortex in hyaluronidase solution (H4272, 1 mg⁄ml) and only those oocytes with a visible first polar body were depleted of the zona pellucida by enzymatic digestion with 1.5 mg/ml pronase (P-8811). Zona-free (ZF) oocytes were stained with 1 μg/ml Hoechst bisbenzimide 33342 (H33342) and enucleated by micromanipulation under UV light. Enucleation was confirmed by direct observation of the stained metaphase plate inside the pipette. Each ooplast was coupled with a MSC using phytohemagglutinin (PHA; L8754) and fused by a double direct current pulse of 1.2 kV/cm, each pulse for 30 μs, separated by 0.1 s. Fused oocytes rested in 5 μl droplets of DMEM/F12 (D8062, Sigma, St Louis, MO, US) for 2.5 h to allow somatic cell reprogramming. Reconstructed embryos were activated with a 4-min incubation in 8.7 μM ionomycin (I24222; Invitrogen, Carlsbad, CA, USA) followed by individual culture in 5 μl droplets of DMEM/F12 with 1 mM 6-dimethylaminopurine (6-DMAP; D2629) and 5 mg/ml cycloheximide (CHX; C7698) for 4 h. Activated embryos were cultured in groups of three in micro-wells similar to the well of the well system [28] into 100 μl droplets of DMEM/F12 supplemented with 10% FBS and 1% ATB, in a humidified gas mixture (5% CO_2_, 5% O_2_, 90% N_2_) at 39°C. Cleavage was evaluated after 4 d of *in vitro* culture and medium was supplemented with 10% FBS. Blastocyst formation was observed between days 7 and 9.

The blastocysts were transferred to synchronized mares as described previously [1]. Briefly, blastocysts were transferred non-surgically in pairs, in 0.5 ml straws with DMEM-F12. Seven to fifteen days after the embryo transfer, pregnancies were diagnosed by transrectal ultrasonography, and fetal monitoring was performed once a week. Three to four weeks before expected parturition, the pregnant mares were transported to an equine hospital (KAWELL, Equine Rehabilitation Center, Solis, Argentina) to give birth.

### Clone genotype identification

Both male and female clones were genotyped with a 15-horse panel of microsatellites, used for routine parentage and identity purposes and standardized according to the International Society of Animal Genetics (ISAG) (https://www.isag.us/committees.asp; S1 Table).

DNA was extracted from 5 hair roots using 100 μL of lysis buffer (10 mM Tris HCl, pH 8.0, 50 mM KCl, 2.5 mM MgCl_2_, 0.5% Tween 20, 10 μg proteinase K; Invitrogen) and incubated at 60°C for 45 min followed by heating at 95°C for 45 min. When a blood sample from the female clone was genotyped, DNA was isolated using the DNeasy Blood & Tissue Kit (Qiagen, Hilden, Germany).

Microsatellites were multiplexed into 2 mixes, as described previously (mix 1: *VHL20*, *HTG4*, *AHT4*, *HMS7*, *ASB17*, *AHT5*, *HMS6*, *ASB23*, *TKY28*; mix 2: *ASB2*, *HMS2*, *HTG10*, *HMS3*, *UCDEQ425*, *TKY325*), grouped by their annealing temperatures –60°C and 56°C, respectively–[29].

Each 25-μL reaction contained 20 ng genomic DNA, 1× PCR buffer, 2.2 mM MgCl_2_, 0.3 mM dNTPs (PCR buffer, MgCl_2_, and dNTPs; Invitrogen), 2% DMSO (dimethylsulfoxide; Millipore, Sigma), 0.15–0.5 μM of each primer (Applied Biosystems, Waltham, MA, US), and 1 U Platinum Taq DNA polymerase (Invitrogen). The PCR protocol consisted of 10 min at 95°C, followed by 31 cycles of 30 s at 95°C, 30 s at appropriate annealing temperature, 30 s at 72°C, and a final extension step of 72°C for 60 min. PCR products were separated by electrophoresis (3130*xl*; Applied Biosystems) and analyzed using GeneMapper v.4.0 (Applied Biosystems).

### Sex molecular analysis

Both hair and blood from the female and the male clones, along with female and male control samples, were tested with a sex panel of X- and Y-linked markers (S2 Table) that were multiplexed into: mix 1 (X-linked microsatellites: *TKY38*, *TKY270*, *Lex0003*, and *Lex026*; Y-linked microsatellite: *ECAYA16*; annealing temperature 50°C), and mix 2 (*AMEL* gene, Y-linked microsatellites: *ECAYH12*, *ECAYM2*, and *SRY* gene; X-linked microsatellites: *Lex0003* and *UCDEQ502*; annealing temperature 58°C) [30, 31]. PCR was carried out and fragments were analyzed as described in the previous section.

### Cytogenetic analysis

The chromosome set was determined according to standard cytogenetic procedures. MSCs and fibroblasts were harvested from cells cultured as described previously. Phytohaemagglutinin stimulated lymphocytes were obtained from peripheral blood and cultured in RPMI-1640 supplemented with 20% FBS [32]. CBG-banding was performed according to the method of Sumner [33].

### FISH analysis

Cell spreads were obtained from the donor MSCs used for the cloning procedure or from peripheral blood of the foals, and prepared as described above. The probes used were EQ-ENX (ZFX—Zinc Finger Protein X-Linked) labelled with rhodamine, and EQ-ENY (TSPY2—Testis Specific Protein Y-Linked 2) labelled with FITC (LEXEL SRL, Buenos Aires, Argentina). Slides were first incubated for 1 h at 45°C. During this time, 1 μl of each probe was mixed with 7μl of hybridization buffer, according to the manufacturer´s instructions. Both mixes were placed on the cell spreads and preparations were then covered and sealed with removable glue. The DNA samples and the probes were simultaneously denatured at 71°C for 8 min, followed by hybridization at 37°C overnight. After hybridization, the glue was carefully removed, and slides were immersed in 2xSSC (0.3M NaCl, 0.03M C_6_H_5_Na_3_O_7_.2H_2_O) at room temperature for 5 min or until the cover glass detached. Preparations were then placed for 2 min in 0.4xSSC + 0.3% Tween a 71°C, and immediately transferred to 2xSSC + 0.1% Tween for 2 min at room temperature. The excess of liquid was removed. Diamino-phenylindole (100ng/ml) and antifade (p-phenylenediamine 1mg/ml) were used as mounting media, and spreads were covered with a cover glass.

Images were taken using an Olympus IX71 epi-fluorescence microscope and Olympus DP-72 camera. Rhodamine and FITC signals were captured in separate images using Cell-Sence Viewer software (Olympus Corporation) and merged using Image-Pro Plus (Media Cybernetics).

## Supporting information

S1 Table Primer sequences for the genotype identification of the cloned foals. (DOCX)

S2 Table Primer sequences of the sex chromosomes molecular analysis. (DOCX)

## References

[1] Olivera R, Moro LN, Jordan R, Luzzani C, Miriuka S, Radrizzani M, et al. In vitro and in vivo development of horse cloned embryos generated with iPSCs, mesenchymal stromal cells and fetal or adult fibroblasts as nuclear donors. PLoS One. 2016;11(10): e0164049. doi: 10.1371/journal.pone.0164049 27732616 PMC5061425

[2] Olivera R, Moro LN, Jordan R, Pallarols N, Guglielminetti A, Luzzani C, et al. Bone marrow mesenchymal stem cells as nuclear donors improve viability and health of cloned horses. Stem Cells Cloning. 2018;11: 13–22. doi: 10.2147/SCCAA.S151763 29497320 PMC5818860

[3] Hinrichs K. A review of cloning in the horse. Reproduction. 2006;52: 398–401.

[4] Inoue K, Ogonuki N, Mekada K, Yoshiki A, Sado T, Ogura A. Sex-reversed somatic cell cloning in the mouse. J Reprod Dev. 2009;55(5): 566–569. doi: 10.1262/jrd.09-099e 19602850

[5] Jeong YH, Lu H, Park CH, Li M, Luo H, Kim JJ, et al. Stochastic anomaly of methylome but persistent SRY hypermethylation in disorder of sex development in canine somatic cell nuclear transfer. Sci Rep. 2016;6(1): 1–11. 27501986 10.1038/srep31088 PMC4977463

[6] Hwang KC, Choi YK, Jeong YI, Park KB, Choi EJ, Jeong YW, et al. Demetylation of the sex-determining region Y gene promoter and incidence of disorder of sex development in cloned dog males. J Physiol Pharmacol. 2020;71(3). doi: 10.26402/jpp.2020.3.05 32991314

[7] Kang JT, Kim HJ, Oh HJ, Hong SG, Park JE, Kim MJ, et al. SRY-positive 78, XY ovotesticular disorder of sex development in a wolf cloned by nuclear transfer. J Vet Sci. 2012;13(2): 211–213. doi: 10.4142/jvs.2012.13.2.211 22705746 PMC3386349

[8] Sugawara A, Goto K, Sotomaru Y, Sofuni T, Ito T. Current status of chromosomal abnormalities in mouse embryonic stem cell lines used in Japan. Comp Med. 2006;56(1): 31–34. 16521857

[9] Estrada JC, Torres Y, Benguria A, Dopazo A, Roche E, Carrera-Quintanar L, et al. Human mesenchymal stem cell-replicative senescence and oxidative stress are closely linked to aneuploidy. Cell Death Dis. 2013;4(6): e691–e691. doi: 10.1038/cddis.2013.211 23807220 PMC3702285

[10] Longo L, Bygrave A, Grosveld FG, Pandolfi PP. The chromosome make-up of mouse embryonic stem cells is predictive of somatic and germ cell chimaerism. Transgenic Res. 1997;6(5): 321–328. doi: 10.1023/a:1018418914106 9322369

[11] Fogh J, Fogh H. Chromosome changes in cell culture induced by mycoplasma infection. Ann N Y Acad Sci. 1973;225(1): 311–329.

[12] Baker DEC, Harrison NJ, Maltby E, Smith K, Moore HD, Shaw PJ, et al. Adaptation to culture of human embryonic stem cells and oncogenesis *in vivo*. Nat Biotechnol. 2007;25: 207–215. 17287758 10.1038/nbt1285

[13] Gorodetsky R, Levdansky L, Gaberman E, Gurevitch O, Lubzens E, McBride WH. Fibrin microbeads loaded with mesenchymal cells support their long-term survival while sealed at room temperature. Tissue Eng Part C Methods. 2011;17(7): 745–755. doi: 10.1089/ten.TEC.2010.0644 21410311 PMC3124111

[14] Raudsepp T, Das PJ, Avila F, Chowdhary BP. The pseudoautosomal region and sex chromosome aneuploidies in domestic species. Sex Dev. 2012;6: 72–83. doi: 10.1159/000330627 21876343

[15] Jiang J, Jing Y, Cost GJ, Chiang JC, Kolpa HJ, Cotton AM, et al. Translating dosage compensation to trisomy 21. Nature. 2013;500(7462): 296–300. doi: 10.1038/nature12394 23863942 PMC3848249

[16] Matsumura H, Tada M, Otsuji T, Yasuchika K, Nakatsuji N, Surani A, et al. Targeted chromosome elimination from ES-somatic hybrid cells. Nat Methods. 2007;4(1): 23–25. doi: 10.1038/nmeth973 17086180

[17] Li LB, Chang KH, Wang PR, Hirata RK, Papayannopoulou T, Russell DW. Trisomy correction in Down syndrome induced pluripotent stem cells. Cell Stem Cell. 2012;11(5): 615–619. doi: 10.1016/j.stem.2012.08.004 23084023 PMC3705773

[18] Zuo E, Huo X, Yao X, Hu X, Sun Y, Yin J, et al. CRISPR/Cas9-mediated targeted chromosome elimination. Genome Biol. 2017;18(1): 1–18. 29178945 10.1186/s13059-017-1354-4 PMC5701507

[19] Kato T, Miyata K, Sonobe M, Yamashita S, Tamano M, Miura K, et al. Production of *Sry* knockout mouse using TALEN via oocyte injection. Sci Rep. 2013;3(1): 1–8. doi: 10.1038/srep03136 24190364 PMC3817445

[20] Song Y, Liu T, Wang Y, Deng J, Chen M, Yuan L, et al. Mutation of the Sp1 binding site in the 5′ flanking region of *SRY* causes sex reversal in rabbits. Oncotarget. 2017;8(24): 38176. doi: 10.18632/oncotarget.16979 28445127 PMC5503524

[21] Kurtz S, Lucas-Hahn A, Schlegelberger B, Göhring G, Niemann H, Mettenleiter TC, et al. Knockout of the HMG domain of the porcine SRY gene causes sex reversal in gene-edited pigs. Proc Natl Acad Sci U S A. 2021;118(2): e2008743118. doi: 10.1073/pnas.2008743118 33443157 PMC7812820

[22] Moro LN, Viale DL, Bastón JI, Arnold V, Suvá M, Wiedenmann E, et al. Generation of myostatin edited horse embryos using CRISPR/Cas9 technology and somatic cell nuclear transfer. Sci Rep. 2020;10(1): 1–10. 32973188 10.1038/s41598-020-72040-4 PMC7518276

[23] Doherty AM, Fisher EM. Microcell-mediated chromosome transfer (MMCT): small cells with huge potential. Mamm Genome. 2003;14(9): 583–592. doi: 10.1007/s00335-003-4002-0 14629108

[24] Paulis M, Susani L, Castelli A, Suzuk T, Hara T, Straniero L, et al. Chromosome transplantation: a possible approach to treat human X-linked disorders. Mol Ther Methods Clin Dev. 2020;17: 369–377. doi: 10.1016/j.omtm.2020.01.003 32099849 PMC7029378

[25] Lasley BL, Bygrave A, Grosveld FG, Pandolfi PP. The limitation of conventional breeding programs and the need and promise of assisted reproduction in nondomestic species. Theriogenology. 1994;41(1): 119–132.

[26] Herrick JR. Assisted reproductive technologies for endangered species conservation: developing sophisticated protocols with limited access to animals with unique reproductive mechanisms. Biol Reprod. 2019;100(5): 1158–1170. doi: 10.1093/biolre/ioz025 30770538

[27] Gambini A, Duque Rodríguez M, Rodríguez MB, Briski O, Flores Bragulat AP, Demergassi N, et al. Horse ooplasm supports *in vitro* preimplantation development of zebra ICSI and SCNT embryos without compromising YAP1 and SOX2 expression pattern. PloS One. 2020;15(9): e0238948. doi: 10.1371/journal.pone.0238948 32915925 PMC7485800

[28] Vajta G, Peura TT, Holm P, Paldi A, Greve T, Trounson AO, et al. New method for culture of zona-included or zona-free embryos: the well of the well (WOW) system. Mol Reprod Dev. 2000;55: 256–264. doi: 10.1002/(SICI)1098-2795(200003)55:3&lt;256::AID-MRD3&gt;3.0.CO;2-7 10657044

[29] Bowling AT, Eggleston-Stott ML, Byrns G, Clark RS, Dileanis S, Wictum E. Validation of microsatellite markers for routine horse parentage testing. Anim Genet. 1997;28: 247–252. doi: 10.1111/j.1365-2052.1997.00123.x 9345720

[30] Kakoi H, Hirota K, Gawahara H, Kurosawa M, Kuwajima M. Genetic diagnosis of sex chromosome aberrations in horses based on parentage test by microsatellite DNA and analysis of X- and Y-linked markers. Equine Vet J. 2005;37: 143–147. doi: 10.2746/0425164054223787 15779627

[31] Martinez MM, Costa M, Ratti C. Molecular screening of XY *SRY*-negative sex reversal cases in horses revealed anomalies in amelogenin testing. J Vet Diagn Invest. 2020;32(6): 938–941. 32865132 10.1177/1040638720952380 PMC7649551

[32] Lear TL, Cox JH, Kennedy GA. Autosomal trisomy in a Thoroughbred colt: 65,XY,+31. Equine Vet J. 1999;31: 85–88. doi: 10.1111/j.2042-3306.1999.tb03796.x 9952335

[33] Sumner AT. A simple technique for demonstrating centromeric heterochromatin. Exp Cell Res. 1972;75: 304–306. doi: 10.1016/0014-4827(72)90558-7 4117921

