# Supplementary material for: First sex modification case in equine cloning
Source: PLoS One. 2023 Jan 4;18(1):e0279869. doi: 10.1371/journal.pone.0279869 (PMC9812313; doi:10.1371/journal.pone.0279869)
Supplement: S1 Table — (DOCX) [file pone.0279869.s001.docx]

**S1 Table. Primer sequences for the genotype identification of the cloned foals.**

| Marker name | Forward sequence | Reverse sequence |
| --- | --- | --- |
| AHT4 | AACCGCCTGAGCAAGGAAGT | CCCAGAGAGTTTACCCT |
| AHT5 | ACGGACACATCCCTGCCTGC | GCAGGCTAAGGAGGCTCAGC |
| ASB2 | CCACTAAGTGTCGTTTCAGAAG | CACAACTGAGTTCTCTGATAGG |
| ASB17 | ACCATTCAGGATCTCCACCG | GAGGGCGGTACCTTTGTACC |
| ASB23 | GAGGGCAGCAGGTTGGGAAGG | ACATCCTGGTCAAATCACAGTCC |
| HMS2 | CTTGCAGTCGAATGTGTATTAAATG | ACGGTGGCAACTGCCAAGGAAG |
| HMS6 | GAAGCTGCCAGTATTCAACCATTG | CTCCATCTTGTGAAGTGTAACTCA |
| HTG4 | CTATCTCAGTCTTGATTGCAGGAC | CTCCCTCCCTCCCTCTGTTCTC |
| VHL20 | CAAGTCCTCTTACTTGAAGACTAG | AACTCAGGGAGAATCTTCCTCAG |
| HTG10 | CCTAATGTCATATGGAAAGCCTTG | TGGGCTTTTTATTCTGATCTGTCACATTT |
| HMS3 | ACATCAGTCAGAAGCTGCGAAC | CCCCTCTTGCTCTAAAGCCCCA |
| HMS7 | TGTTSTTGAAACATACATTGACTGT | CAGGAAACTCATGTTGATACCATC |
| UCDEQ425 | AGCTGCCTCGTTAATTCA | CTCATGTCCGCTTGTCTC |
| TKY325 | GGATGGAGTGAGATAATACC | TGGATGAACCATGAATAGTG |
| TKY28 | TTCAGCAGGGTCTCATGCCAC | TTCGGCTCTGGTTCAAGAGG |
