# Supplementary material for: First sex modification case in equine cloning
Source: PLoS One. 2023 Jan 4;18(1):e0279869. doi: 10.1371/journal.pone.0279869 (PMC9812313; doi:10.1371/journal.pone.0279869)
Supplement: S2 Table — (DOCX) [file pone.0279869.s002.docx]

**S2 Table. Primer sequences of the sex chromosomes molecular analysis.**

| Marker name | Forward sequence | Reverse sequence |
| --- | --- | --- |
| LEX026 | TCCAGAGTGAATGGCAAATC | AATATCTTGCTAATCTCATA |
| LEX003 | ACATCTAACCAGTGCTGAGACT | AAGAACTAGAACCTACAACTAGG |
| TKY38 | TAAGTATTCTCATAAACGGG | GGAATAATAACAGCATCCTC |
| TKY270 | CTGCTTTAGAGAAACAAACT | CCATGGTGAGAAAAATGAGA |
| UCDEQ502 | AGAGGGCAAAGTCAGAGCTT | AGCACCTGATGCTTCTTGTT |
| ECAYH12 | CGAACAGGTGACGAAGCATC | GCAGACATGCACACCAACC |
| ECAYM2 | TGGTTCAGATGGTGTATTTTGTT | TTTGCAGCCAGTACCTACCTT |
| ECAYA16 | TGACTGGAAATTGAAGATG | TTGTAGCAACAAAGTAACAC |
| SRY | TGCATTCATGGTGTGGTCTCGTG | AGTCTGCGGAAGCGGTTTGTCA |
| AMEL | CCAACCCAACACCACCAGCCAAACCTCCCT | AGCATAGGGGGCAAGGGCTGCAAG GGGAAT |
